# Giant cell tumor of talus: a case report

**DOI:** 10.1186/1757-1626-2-74

**Published:** 2009-01-21

**Authors:** Siddhartha Sharma, Iftikhar H Wani, Nital Gupta, Nirdosh Mahajan, Abdul Q Salaria

**Affiliations:** 1 Department of Orthopedics, Government Medical College, Jammu, India

## Abstract

Giant cell tumor of talus is a rare entity. In contrast to GCT of long bones, most cases occur in a younger age group and tend to be multicentric. The authors report a case of GCT in a 19 year old boy which had led to extensive destruction of the talus. In view of the extensive involvement, total talectomy along with tibio – calcaneal arthrodesis was performed. At 6 months of followup, the patient had a painless and well arthrodesed ankle. There was no evidence of recurrence at 18 months of followup.

## Background

Talus is a rare site for involvement by Giant Cell Tumor. The authors report a Giant Cell Tumor which had led to destruction of the entire talus in a 19 year old boy. In view of the extensive involvement, total talectomy along with tibio – calcaneal arthrodesis was performed with the aim of achieving a stiff but painless joint.

## Case presentation

A 19 year old boy presented with chief complaints of insidious onset pain in the right ankle since the last two years, swelling in the right ankle since the last six months and inability to bear weight on right side since the last six months. There was no history of fever, loss of appetite, loss of weight, similar complaints in other joints or history of similar complaints in the past. The family, occupational, recreational and drug histories were not significant. The general physical and systemic examinations were within normal limits.

On local examination, the attitude of the limb was neutral. There was a 5 × 4 cm swelling over dorsum of right foot and anterior aspect of ankle joint. There were no visible veins, sinus or discharge from the swelling. The local temperature was increased and the swelling was tender. All movements at the ankle joint were painfully restricted.

**Figure 1:**
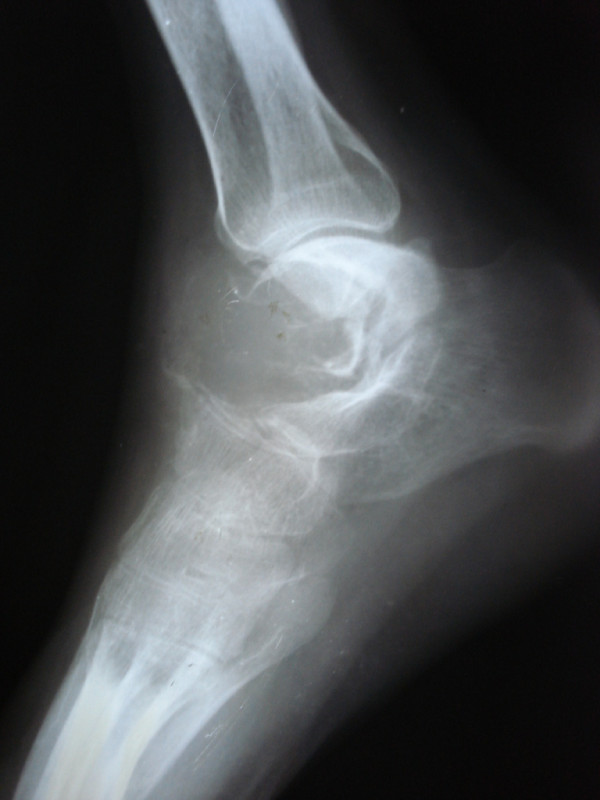
**Lateral radiograph of ankle showing a radiolucent lesion occupying the talus with expansion and thinning of the cortex**.

**Figure 2:**
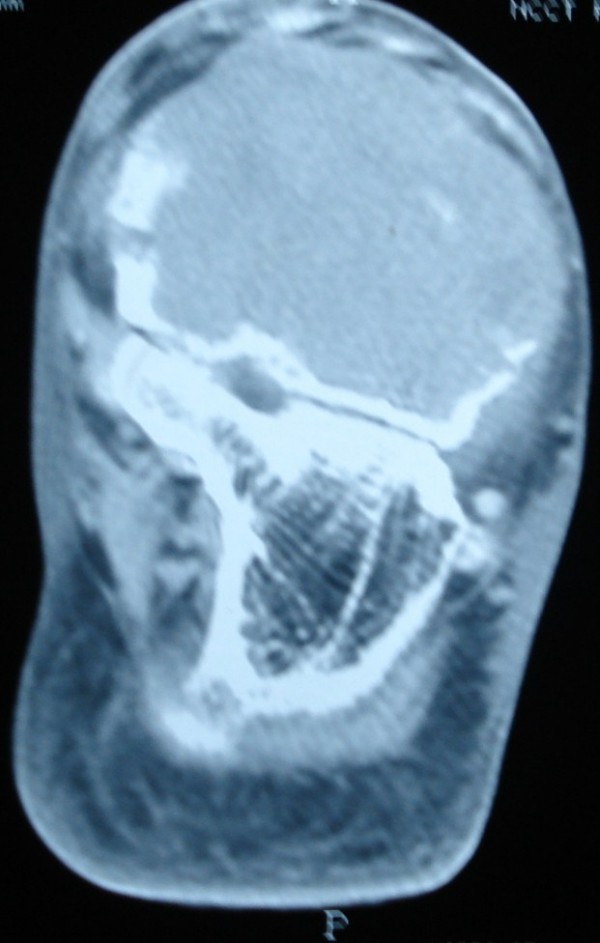
**CT scan of ankle showing an expansile lytic lesion in the talus causing cortical destruction and extending into soft tissues**.

Serum biochemistry studies were within normal limits. AP and lateral radiographs of the ankle showed a radiolucent lesion occupying the whole of talus with expansion and thinning of the cortex (Figure 1). CT scan revealed an expansile soft tissue mass in the talus causing cortical destruction and extension into soft tissues. The joint space between calcaneum and talus was well preserved (Figure 2). A Fine Needle Aspiration Cytology study of the swelling was done and a provisional diagnosis of Giant Cell Tumor was made.

The condition, its prognosis and various treatment modalities were discussed at length with the patient. In view of the extensive involvement of talus, total talectomy with tibio – calcaneal arthrodesis was planned. The patient was a manual laborer and therefore chose the option of a stiff but painless joint.

**Figure 3:**
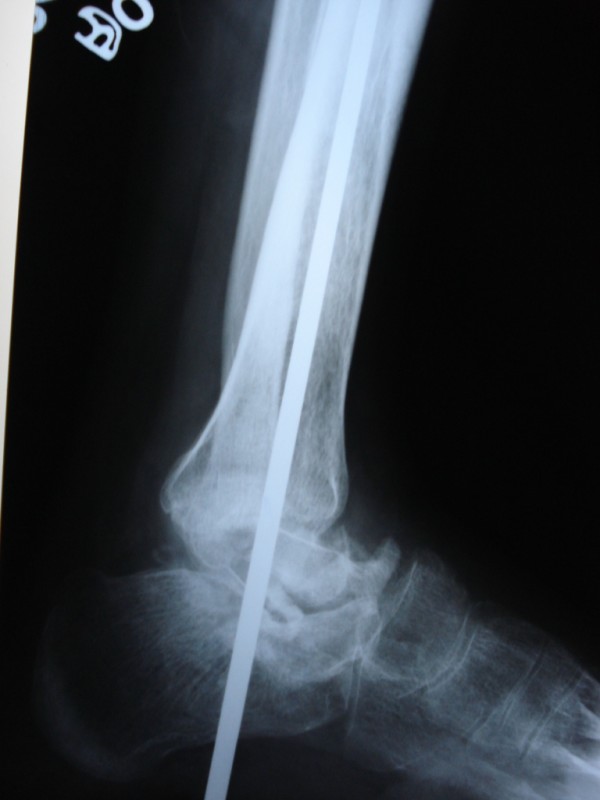
**Immediate postoperative radiograph showing talectomy, bone grafting and stabilization with a Steinmann pin**.

**Figure 4:**
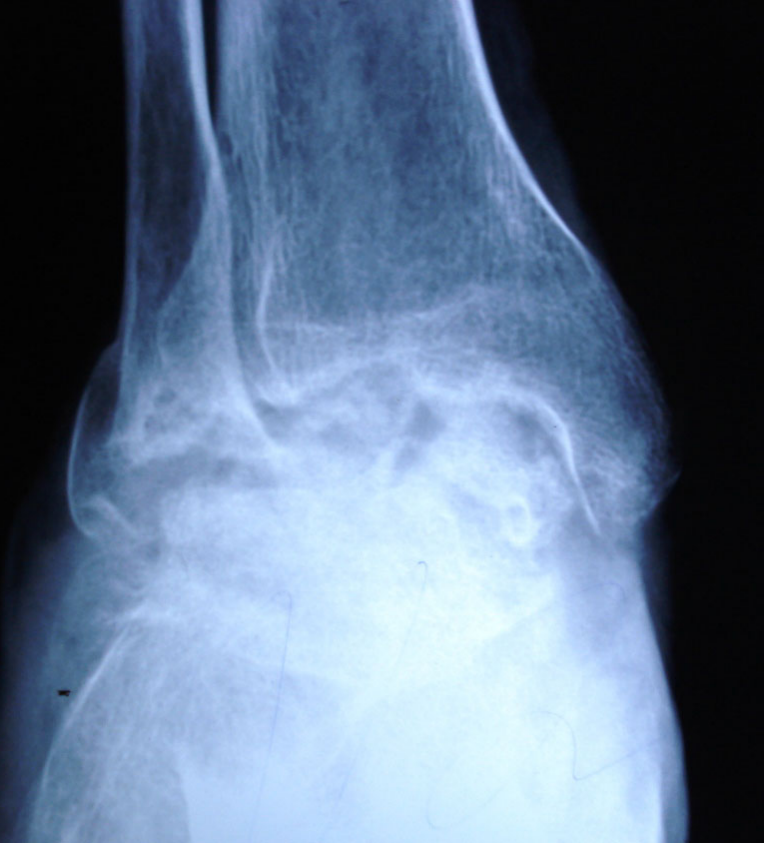
**Radiograph at 6 months follow-up showing a well arthrodesed ankle**.

Total talectomy was performed via the standard anterior approach. Fusion was achieved by autologous iliac crest graft and stabilization with a Steinmann pin (Figure 3). Histopathological examination of the talectomy material confirmed the diagnosis of Giant Cell Tumor. The patient was advised not to bear weight on the affected limb for 8 weeks and mobilized in a short leg walking cast thereafter. The Steinmann pin and cast were removed after 4 months. At 6 months of follow-up, the patient had a smooth healed scar with a painless and well arthrodesed ankle with no evidence of recurrence (Figure 4). There was no evidence of recurrence at 18 months of followup.

## Discussion

Giant cell tumour, also known as osteoclastoma, is a fairly common bone tumour accounting for 5% of all the primary bone tumours. It is a benign tumour with a tendency for local aggressiveness and high chances of recurrence. The most common sites are distal end of femur, upper end of tibia and lower end of radius [1]. The foot is an unusual site of presentation and GCTs involving hand and foot bones appear to occur in a younger age group and tend to be multicentric [2].

The clinical picture is that of insidious onset pain, which in many cases may be mismanaged as ankle sprain. A history of preceding trivial trauma may be present. Other features are non specific.

Radiologically, the tumour appears as an eccentric lytic lesion with cortical thinning and expansion. There is absence of reactive new bone formation. The tumour may erode the cortex and invade the joint. Pathological fracture may also be seen [3]. CT scanning permits accurate delineation of the tumour extent and helps in deciding the line of management i.e. Curettage Vs Talectomy.

Many authors have reported satisfactory results with intralesional curettage and bone grafting [4]. However, curettage alone has a high rate of recurrence and adjuvants like Methylmethacrylate (bone cement), Cryotherapy and Phenol have been suggested.

Partial or total talectomy may be contemplated in cases where there is extensive involvement of the talus. Arthrodesis may or may not be done, but it is said that arthrodesis is essential after resection of all tarsal bones except calcaneum [5]. Fresh frozen osteochondral allograft reconstruction has also been described for an aggressive GCT of talus but there is paucity of literature on this particular modality of treatment [6]. The trend is towards limb salvage and amputation is reserved for recurrences and only rarely done.

## Abbreviations

AP: Antero Posterior; CT Scan: Computed Tomographic; Scan GCT: Giant Cell Tumor.

## Consent

Written informed consent was obtained from the patient for publication of this case report and accompanying images. A copy of the written consent is available for review by the Editor-in-Chief of this journal.

## Competing interests

The authors declare that they have no competing interests.

## Authors' contributions

IHW analyzed and interpreted the patient data regarding the disease. NM and NG discussed the case with radiology and pathology experts and formulated the treatment plan. AQS, SS, NM and NG performed the operative procedure. SS was responsible for followup and prepared the manuscript. All authors read and approved the final manuscript.
